# The Impact of Left Ventricle Ejection Fraction Reduction and Transient Ischemic Dilation in Patients With Normal Single-Photon Emission Computed Tomography (SPECT) Myocardial Perfusion Imaging

**DOI:** 10.7759/cureus.32950

**Published:** 2022-12-26

**Authors:** Paulo Medeiros, Bárbara Pereira, Jorge Rodrigues

**Affiliations:** 1 Cardiology, Hospital de Braga, Braga, PRT; 2 Nuclear Medicine, Centro Hospitalar Universitário de São João, Porto, PRT; 3 Oncology, Hospital de Braga, Braga, PRT

**Keywords:** invasive coronary angiography, transient ischemic dilation, left ventricle ejection fraction, single-photon emission positron tomography, coronary artery disease

## Abstract

Introduction: Coronary artery disease (CAD) is a leading cause of death in developed countries. Non-invasive functional imaging modalities are currently recommended as initial diagnostic tests in patients with an intermediate-high pretest probability of CAD. Single-photon emission computed tomography myocardial perfusion imaging (SPECT-MPI) creates images of regional myocardial tracer uptake, reflecting relative myocardial blood flow. However, there are other non-perfusion predictors of CAD, such as transient ischemic dilatation (TID) and reduced post-stress left ventricle ejection fraction (LVEF). Available data regarding these parameters is controversial. The aim of our study was to evaluate the incidence of significant CAD in patients with non-perfusion high-risk markers of ischemia despite a normal SPECT-MPI.

Methods: Single-center, observational, retrospective, and longitudinal study. Inclusion criteria were age ≥18 years, normal SPECT-MPI, and availability of gated study for LVEF and volume analysis. Exclusion criteria were any known cardiomyopathy or congenital heart disease and known CAD. Non-perfusion high-risk markers: LVEF reduction ≥5% on post-stress images; TID (defined as a stress/rest left ventricle volume ratio ≥ 1.15), including end-systolic, end-diastolic, and mean volumes. The primary endpoint was the identification of significant CAD (stenosis >70% on an epicardial coronary artery or >50% on the left main artery) on invasive coronary angiography.

Results: A total of 197 patients met the inclusion criteria. Mean age was 64 ± 12.6 years and 59.4% (n = 117) of patients were male. Overall, 26% of patients had LVEF reduction ≥5% on stress study; 24.9% had a stress/rest end-systolic volume ratio ≥ 1.15; 7.1% had a stress/rest mean volume ratio ≥ 1.15; 7.1% had a stress/rest end-diastolic volume ratio ≥ 1.15. Time-to-primary endpoint was significantly lower in patients with LVEF reduction ≥5% on stress study (67.99 (95% CI 60.49-75.49) vs. 77.56 months (95% CI 75.14-79.99); p = 0.003) and in patients with stress/rest end-systolic volume ratio ≥ 1.15 (68.39 (95% CI 60.69-76.10) vs. 77.31 months (95% ICCI 74.68-79.76); p = 0.013).

Conclusion: In patients with normal perfusion on SPECT-MPI, the incidence of significant CAD was significantly higher in those with LVEF reduction ≥ 5% on stress study and in those with a stress/rest end-systolic volume ratio ≥ 1.15, during a follow-up period of five years.

## Introduction

Coronary artery disease (CAD) is a leading cause of death in developed countries, being also a source of significant healthcare-associated costs. Non-invasive functional imaging modalities, such as stress echocardiography, stress cardiac magnetic resonance, single-photon emission computed tomography (SPECT), or positron emission tomography are currently recommended as initial diagnostic tests in patients with an intermediate-high pretest probability of CAD [1].

SPECT myocardial perfusion imaging (SPECT-MPI) creates images of regional myocardial tracer uptake, reflecting relative myocardial blood flow (at rest and during stress), and also providing information regarding the location, extent, and reversibility of perfusion defects [2]. This technique is an important gatekeeper for invasive coronary angiography (ICA) and may provide important information regarding the need for revascularization [3].

Perfusion is the most extensively reported outcome of SPECT. However, there are other non-perfusion predictors of CAD, such as transient ischemic dilatation (TID) and reduced post-stress left ventricle ejection fraction (LVEF). TID is defined as the apparent presence of left ventricular dilation on post-stress relative to rest images; it has been associated with worse cardiovascular outcomes in patients with abnormal SPECT-MPI but has also been linked to balanced ischemia in patients with otherwise normal SPECT-MPI [4-6]. However, data regarding TID in patients with normal SPECT-MPI is controversial. Similarly, although a reduced LVEF in post-stress images has been associated with severe CAD, the literature is also controversial.

The aim of our study was to evaluate the incidence of significant CAD in patients with non-perfusion high-risk markers of ischemia despite a normal SPECT-MPI.

## Materials and methods

Study design and patients

This was a single-center, observational, retrospective, and longitudinal study. Patients were included according to the following criteria: age ≥18 years, normal SPECT-MPI, and availability of gated study for LVEF and volume analysis. Defined exclusion criteria were any known cardiomyopathy or congenital heart disease and known CAD. Outcome data were retrospectively collected in a follow-up period of five years.

Cardiovascular risk factors

The considered cardiovascular risk factors definitions were established according to the current guidelines: hypertension was defined by a systolic blood pressure of ≥140 mmHg, diastolic blood pressure of ≥90 mmHg or use of antihypertensive medication; dyslipidemia was defined as total cholesterol ≥200 mg/dL or use of lipid-lowering medication; diabetes mellitus was defined as hemoglobin A1c levels of ≥6.5% or use of antidiabetic treatment; smoking status was considered only if the patient was currently a smoker. Chronic kidney disease was stated if the glomerular filtration rate was <60 mL/min (stage 3 or higher).

SPECT imaging protocol and definitions

Anti-ischemic drugs were withdrawn before the procedure (48 h for beta-blockers and calcium channel blockers and 6 h for nitrates). The radiotracer was technetium-99m sestamibi. Under continuous electrocardiographic monitoring, treadmill exercise test or adenosine perfusion were used prior to performing gated stress image acquisition. Rest images were acquired in a second moment (same day or a week later).

The 17-segment model was used as a semi-quantitative measurement of radiotracer uptake (0 = normal, 1 = equivocal, 2 = moderate, 3 = severe reduction of uptake, and 4 = absence of detectable tracer). Summed stress score (SSS) was obtained by adding the values of each segment. Normal SPECT-MPI was defined as SSS < 4.

Three-dimensional left ventricle volumes and ejection fractions were derived from local software algorithms. After consulting the available literature, we considered the following two non-perfusion high-risk markers: LVEF reduction ≥5% on post-stress images; TID (defined as a stress/rest left ventricle volume ratio ≥ 1.15), including end-systolic, end-diastolic, and mean volumes [7-9].

Primary endpoint

Primary endpoint was the identification of significant CAD on ICA (stenosis >70% on an epicardial coronary artery or >50% on the left main artery). We compared the incidence of the primary endpoint in patients with and without any of the defined non-perfusion high-risk markers.

Statistical analysis

Continuous variables were expressed as the mean ± standard deviation. The unpaired student’s t-test (for normally distributed variables) or the Wilcoxon rank-sum test (for non-parametrically distributed variables) were used to compare groups. Categorical variables were compared using Pearson Chi-squared tests or Fisher exact test for cell counts <6. Kaplan-Meier estimates and long-rank test were used to determine event-free survival and compare groups, respectively.

All statistical analyses were performed using SPSS (Version 28 IBM Corp, Armonk, NY) for Windows. A p-value <0.05 were considered statistically significant and only two-sided tests were conducted.

## Results

Baseline characteristics

A total of 197 patients met the inclusion criteria. Baseline characteristics are described in Table 1. Mean age was 64 ± 12.6 years and 59.4% (n = 117) of patients were male. Considering cardiovascular risk factors, 75.6% (n = 149) of patients had hypertension, 54.3% (n = 107) had dyslipidemia, 26.9% (n = 53) had diabetes mellitus, and 14.2% (n = 28) had smoking habits. Also, 14.2% (n = 28) had chronic kidney disease.

**Table 1:** Baseline characteristics of the population LV: left ventricle; LVEDV: left ventricle end-diastolic volume; LVEF: left ventricle ejection fraction; LVESV: left ventricle end-systolic volume

Table 1. Baseline characteristics (n = 197)	
Male gender	80 (40.6%)
Mean age, years ± SD	64 ± 12.6
Hypertension, n (%)	149 (75.6%)
Dyslipidemia, n (%)	108 (54.3%)
Diabetes mellitus, n (%)	53 (26.9%)
Smoking, n (%)	28 (14.2%)
Chronic kidney disease, n (%)	28 (14.2%)
LVEF reduction >5% on stress, n (%)	52 (26.4%)
LVEDV stress/rest ratio > 1.15, n (%)	14 (7.1%)
LVESV stress/rest ratio > 1.15, n (%)	49 (24.9%)
Mean LV volume stress/rest ratio > 1.15, n (%)	14 (7.1%)

SPECT volumetric features

Regarding resting LV systolic function, 11.7% of patients had a baseline LVEF < 50%. Overall, 26.4% of patients had LVEF reduction ≥5% on stress study; 24.9% had a stress/rest end-systolic volume ratio ≥ 1.15; 7.1% had a stress/rest mean volume ratio ≥ 1.15; 7.1% had a stress/rest end-diastolic volume ratio ≥ 1.15.

Time-to-primary endpoint

**Figure 1:**
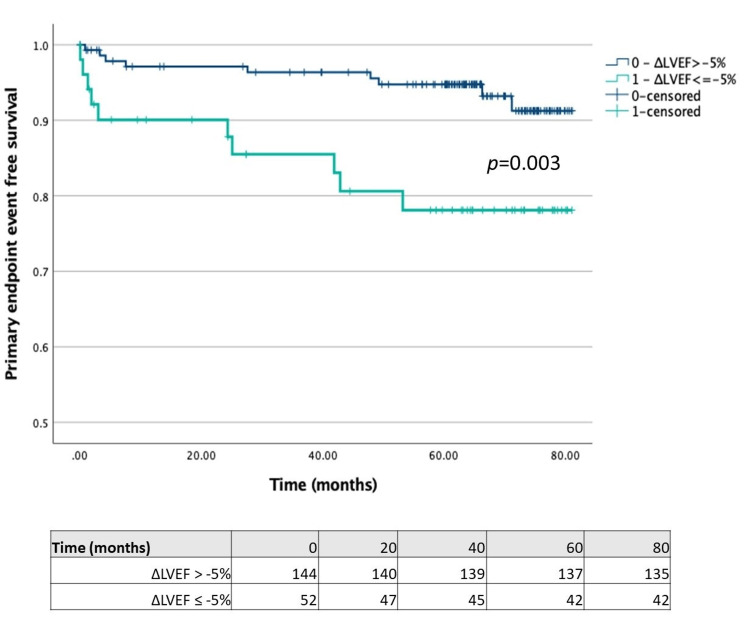
Kaplan-Meier plot for LVEF reduction ≥ 5% LVEF: left ventricle ejection fraction

**Figure 2:**
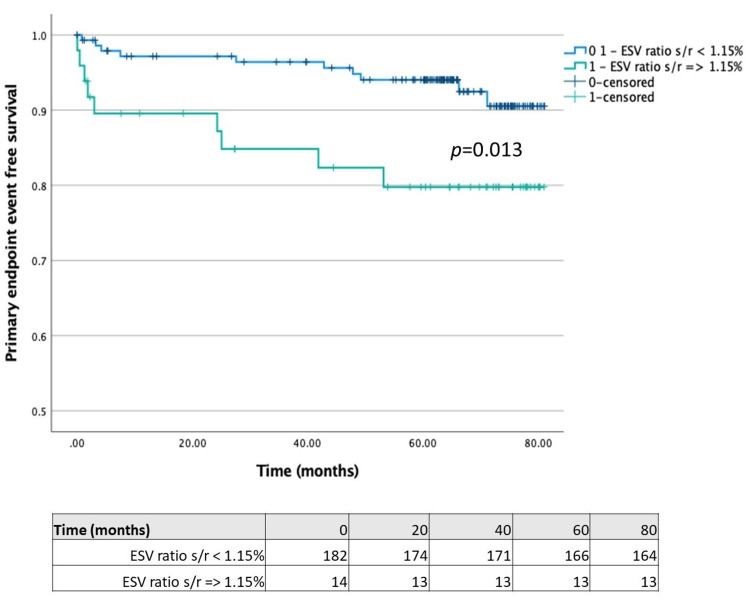
Kaplan-Meier plot for LVESV stress/rest ratio ≥1.15 LVESV: left ventricle end-systolic volume

**Figure 3:**
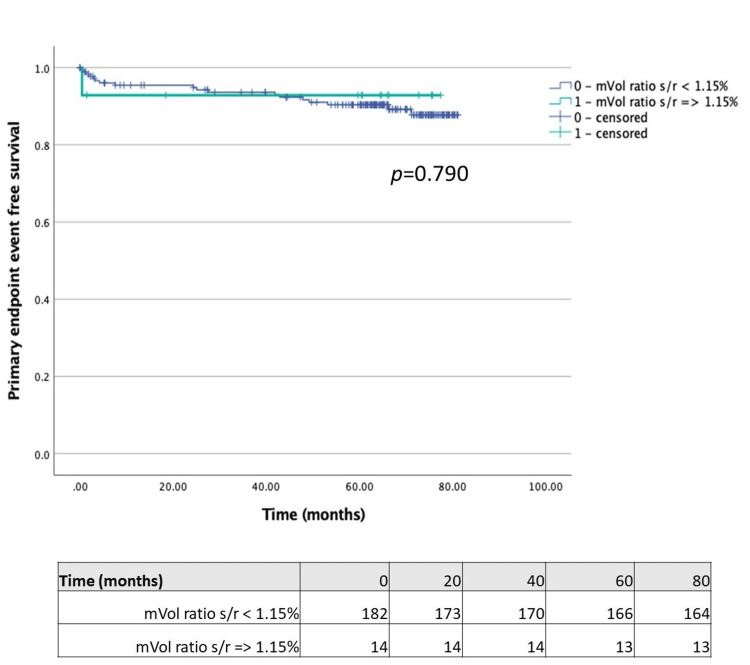
Kaplan-Meier plot for mean LV volume stress/rest ratio ≥1.15 LV: left ventricle

**Figure 4:**
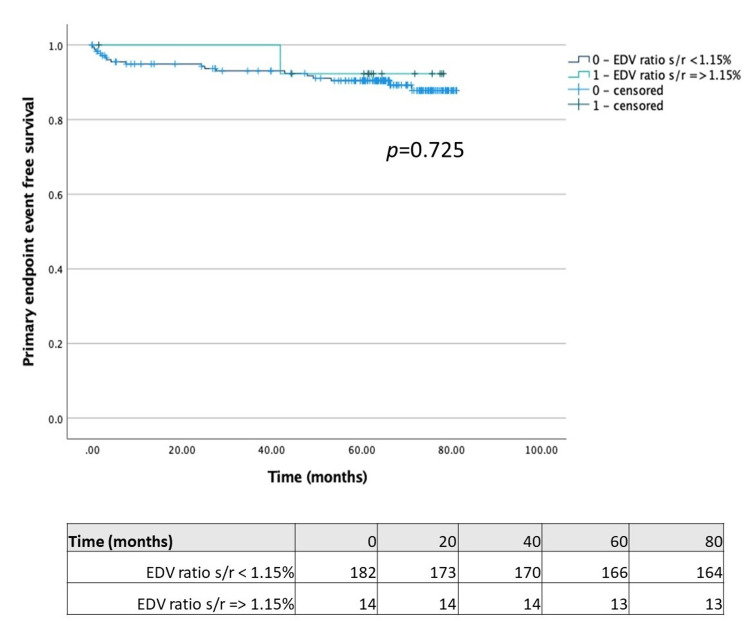
Kaplan-Meier plot for LVEDV stress/rest ratio ≥1.15 LVEDV: left ventricle end-diastolic volume

Comparing patients with LVEF reduction ≥5% on stress study with those without this feature (Figure 1), we found that the former had a significantly lower time-to-primary endpoint (67.99 (95% CI 60.49-75.49) vs. 77.56 months (95% CI 75.14-79.99); p = 0.003). Regarding TID, we separately compared patients with each of the different volume measures (stress/rest mean volume ratio ≥ 1.15, stress/rest end-systolic volume ratio ≥ 1.15 and stress/rest end-diastolic volume ratio ≥ 1.15) with those without the respective features. Although the time-to-primary endpoint was significantly lower in patients with stress/rest end-systolic volume ratio ≥ 1.15 compared with patients without this feature (68.39 (95% CI 60.69-76.10) vs. 77.31 months (95% ICCI 74.68-79.76); p = 0.013) (Figure 2), the same was not observed in patients with stress/rest mean or end-diastolic volume ratios ≥ 1.15 (Figures 3-4).

## Discussion

Normal SPECT-MPI has been associated with low rates of cardiac death and non-fatal myocardial infarction (<1%/year) [10]. A recent study by Kassab et al. stated that, compared with patients with normal SPECT-MPI (SSS = 0), patients with near normal perfusion (SSS 1-3) had a higher rate of obstructive CAD, although no difference was found in the incidence of hard cardiac events [11]. The purpose of our study was to evaluate the incidence of significant CAD in patients with non-perfusion high-risk markers of ischemia, namely LVEF reduction on stress study and TID, despite a normal SPECT-MPI.

In our study, LVEF reduction ≥5% was associated with a lower time until the identification of significant CAD on ICA. In individuals devoid of CAD, the normal response to exercise of vasodilator stress should be maintenance or increase in LVEF [12]. Myocardial stunning or severe subendocardial ischemia have been suggested as possible mechanisms behind the post-stress reduction in LVEF [13]. Conflicting results are published regarding the significance of this parameter. For a long time, numerous studies have correlated LVEF reduction with significant CAD on ICA, both in exercise and vasodilator protocols, although most of the featured patients had also abnormal SPECT-MPI [14-17]. In contrast, a study by Gomez et al. concluded that LVEF reduction following regadenoson stress did not predict severe/significant CAD, regardless of MPI findings [7].

Even though the pathophysiology of TID is still in debate, several papers have addressed this topic. In our study, we evaluated stress/rest ratios in end-systolic, end-diastolic, and mean volumes; only TID defined by end-systolic volumes was significantly associated with a lower time-to-primary endpoint. Once again, conflicting results have been published. On one hand, a work by Abidov et al. reported an independent and incremental risk of cardiac events in patients with higher levels of CAD [6]. On the other, Valdiviezo et al. [18] concluded that TID in a normal SPECT-MPI was not associated with severe CAD on ICA; these results were supported by Halligan et al. [4] in a study using correlation with coronary computed tomography angiography.

Our study has several limitations. First, it is a retrospective single-center study, and our population may not represent other realities. Second, the cut-offs used to create the groups were those reported by the available literature. Third, the compared groups were not matched, and the number of individuals in each group is asymmetrical, which may be a source of bias. Also, our endpoint was the identification of significant coronary stenosis on ICA during a follow-up period of five years; during follow-up, the patients that performed ICA likely experienced ischemic symptoms or had an unexplained decrease in LVEF; asymptomatic patients did not perform ICA so that we cannot exclude the presence of asymptomatic but significant coronary lesions. Although we found our results interesting, larger and prospective studies are still needed to further clarify the role of non-perfusion high-risk markers in patients with otherwise normal SPECT-MPI.

## Conclusions

In patients with normal perfusion on SPECT-MPI, the incidence of significant CAD was significantly higher in those with LVEF reduction ≥ 5% on stress study and in those with a stress/rest end-systolic volume ratio ≥ 1.15, during a follow-up period of five years.

These markers may be of clinical relevance in the follow-up of patients with suspected CAD.

## References

[1] Knuuti J, Wijns W, Saraste A (2020). 2019 ESC Guidelines for the diagnosis and management of chronic coronary syndromes. Eur Heart J.

[2] Verberne HJ, Acampa W, Anagnostopoulos C (2015). EANM procedural guidelines for radionuclide myocardial perfusion imaging with SPECT and SPECT/CT: 2015 revision. Eur J Nucl Med Mol Imaging.

[3] Hachamovitch R, Hayes SW, Friedman JD, Cohen I, Berman DS (2003). Comparison of the short-term survival benefit associated with revascularization compared with medical therapy in patients with no prior coronary artery disease undergoing stress myocardial perfusion single photon emission computed tomography. Circulation.

[4] Halligan WT, Morris PB, Schoepf UJ (2014). Transient ischemic dilation of the left ventricle on SPECT: correlation with findings at coronary CT angiography. J Nucl Med.

[5] McClellan JR, Travin MI, Herman SD (1997). Prognostic importance of scintigraphic left ventricular cavity dilation during intravenous dipyridamole technetium-99m sestamibi myocardial tomographic imaging in predicting coronary events. Am J Cardiol.

[6] Abidov A, Bax JJ, Hayes SW (2003). Transient ischemic dilation ratio of the left ventricle is a significant predictor of future cardiac events in patients with otherwise normal myocardial perfusion SPECT. J Am Coll Cardiol.

[7] Gomez J, Golzar Y, Fughhi I, Olusanya A, Doukky R (2018). The significance of post-stress decrease in left ventricular ejection fraction in patients undergoing regadenoson stress gated SPECT myocardial perfusion imaging. J Nucl Cardiol.

[8] Lette J, Bertrand C, Gossard D (1995). Long-term risk stratification with dipyridamole imaging. Am Heart J.

[9] Alama M, Labos C, Emery H, Iwanochko RM, Freeman M, Husain M, Lee DS (2018). Diagnostic and prognostic significance of transient ischemic dilation (TID) in myocardial perfusion imaging: a systematic review and meta-analysis. J Nucl Cardiol.

[10] Bourque JM, Beller GA (2011). Stress myocardial perfusion imaging for assessing prognosis: an update. JACC Cardiovasc Imaging.

[11] Kassab K, Hussain K, Torres A, Iskander F, Iskander M, Khan R, Doukky R (2022). The diagnostic and prognostic value of near-normal perfusion or borderline ischemia on stress myocardial perfusion imaging. J Nucl Cardiol.

[12] Ababneh AA, Sciacca RR, Kim B, Bergmann SR (2000). Normal limits for left ventricular ejection fraction and volumes estimated with gated myocardial perfusion imaging in patients with normal exercise test results: influence of tracer, gender, and acquisition camera. J Nucl Cardiol.

[13] Hida S, Chikamori T, Tanaka H (2009). Diagnostic value of left ventricular function after adenosine triphosphate loading and at rest in the detection of multi-vessel coronary artery disease using myocardial perfusion imaging. J Nucl Cardiol.

[14] Paul AK, Hasegawa S, Yoshioka J, Mu X, Maruyama K, Kusuoka H, Nishimura T (2002). Characteristics of regional myocardial stunning after exercise in gated myocardial SPECT. J Nucl Cardiol.

[15] Toba M, Kumita S, Cho K, Ibuki C, Kumazaki T, Takano T (2004). Usefulness of gated myocardial perfusion SPECT imaging soon after exercise to identify postexercise stunning in patients with single-vessel coronary artery disease. J Nucl Cardiol.

[16] Dorbala S, Vangala D, Sampson U, Limaye A, Kwong R, Di Carli MF (2007). Value of vasodilator left ventricular ejection fraction reserve in evaluating the magnitude of myocardium at risk and the extent of angiographic coronary artery disease: a 82Rb PET/CT study. J Nucl Med.

[17] Van Tosh A, Votaw JR, Reichek N, Palestro CJ, Nichols KJ (2013). The relationship between ischemia-induced left ventricular dysfunction, coronary flow reserve, and coronary steal on regadenoson stress-gated (82)Rb PET myocardial perfusion imaging. J Nucl Cardiol.

[18] Valdiviezo C, Motivala AA, Hachamovitch R (2011). The significance of transient ischemic dilation in the setting of otherwise normal SPECT radionuclide myocardial perfusion images. J Nucl Cardiol.

